# A Novel Strain of the Cyanobacterial Growth-promoting Bacterium, *Rhodococcus* sp. AF2108, Enhances the Growth of *Synechococcus elongatus*

**DOI:** 10.1264/jsme2.ME24050

**Published:** 2024-12-28

**Authors:** Pei Yu Tan, Yuta Kato, Masaaki Konishi

**Affiliations:** 1 Graduate School of Engineering, Kitami Institute of Technology, 165 Koen-cho, Kitami, Hokkaido 090–8507, Japan; 2 Kankyo Daizen Co., Ltd., 438–7, Tanno-cho 3-ku, Kitami, Hokkaido, 099–2103, Japan; 3 Department of Applied Chemistry, Kitami Institute of Technology, 165 Koen-cho, Kitami, Hokkaido, 090–8507, Japan

**Keywords:** co-culture, chlo­rophyll, cyanobacteria, phytohormone, siderophore

## Abstract

To enhance the growth of the cyanobacterium *Synechococcus elongatus*, the present study conducted direct screening for cyanobacterium growth-promoting bacteria (CGPB) using co-cultures. Of the 144 strains obtained, four novel CGPB strains were isolated and phylogenetically identified: *Rhodococcus* sp. AF2108, *Ancylobacter* sp. GA1226, *Xanthobacter* sp. AF2111, and *Shewanella* sp. OR151. A co-culture of *S. elongatus* with the most effective CGPB strain, *Rhodococcus* sp. AF2108, achieved a 8.5-fold increase in the chlo­rophyll content of cyanobacterial cells over that in a monoculture. A flow cytometric ana­lysis showed a 3.9-fold increase in the number of *S. elongatus* cells in the co-culture with *Rhodococcus* sp. AF2108. These results were attributed to increases in forward scattering and chlo­rophyll fluorescence intensities. The new *Rhodococcus* strain appears to be one of the most effective CGPBs described to date.

*Cyanobacteria*, as photoautotrophic prokaryotes, require only sunlight, carbon dioxide (CO_2_), water, and minimal nutrients for growth, thereby eliminating the need for expensive carbon sources and complex growth media (Singh and Dhar, 2019). *Cyanobacteria* have been employed to capture and convert CO_2_ into other compounds, such as ethanol (Liang *et al.*, 2018), isopropanol (Hirokawa *et al.*, 2015), and fatty acids (Ruffing, 2014). However, further enhancements in the productivity of cyanobacteria for economic feasibility are currently limited by their growth rates. Therefore, strategies that enhance cyanobacterial proliferation have potential in the further development of cyanobacteria-based bioprocesses.

Co-cultivations with symbiont bacteria may promote the proliferation and metabolic synthesis of cyanobacteria via the exchange of metabolites, molecules, or nutrients through complex mechanisms. A previous study reported that the cyanobacterium *Synechocystis* sp. PCC 6803 degraded phenanthrene and also increased the cyanobacterial content of chlo­rophyll 8-fold when co-cultured for 20 days with the aerobic heterotrophic bacterium *Pseudomonas* sp. GM41 (Abed, 2010). These findings indicate the utility of technique for bioremediating oil-polluted sites that circumvents the high costs associated with the use of organic and inorganic fertilizers (Abed, 2010). Symbiont co-cultivation may also reduce the use of exogeneous nutrients because a cyanobacterium and its symbiont working together may efficiently exchange remineralized compounds, thereby increasing nutrient cycling and reducing the discharge of wastewater (Wu *et al.*, 2023). Therefore, a co-culture approach may be an eco-friendly alternative for enhancing biomass production.

In the present study, we performed symbiont co-culture screening for the cyanobacterium *S. elongatus* using bacterial isolates derived from agricultural wastewater. We obtained, taxonomically identified, and characterized novel cyanobacterium growth-promoting bacteria (CGPBs).

## Materials and Methods

### Isolation of CGPBs

CGPBs were isolated from agriculture wastewater derived from cattle urine by a simplified activated sludge treatment. Agriculture waste has been used as a liquid fertilizer via phytohormone-like responses in *Arabidopsis thaliana* (Kato and Konishi, 2024). With the aim of increasing the variety of isolated species, six different isolation media were prepared for use in the present study: JCM medium number 520 (JCM 520) agar (2.0% [w/v] agar) (Biebl and Pfennig, 1981), consisting of 0.5‍ ‍g‍ ‍L^–1^ KH_2_PO_4_, 0.25‍ ‍g‍ ‍L^–1^ CaCl_2_·2H_2_O, 3.0‍ ‍g‍ ‍L^–1^ MgSO_4_·7H_2_O, 0.68‍ ‍g‍ ‍L^–1^ NH_4_Cl, 20‍ ‍g‍ ‍L^–1^ NaCl, 3.0‍ ‍g‍ ‍L^–1^ sodium malate, 3.0‍ ‍g‍ ‍L^–1^ sodium pyruvate, 0.4‍ ‍g‍ ‍L^–1^ yeast extract, 5‍ ‍mg L^–1^ ferric citrate, 2‍ ‍mg L^–1^ vitamin B12, 70‍ ‍μg L^–1^ ZnCl_2_·5H_2_O, 100‍ ‍μg L^–1^ MnCl_2_·4H_2_O, 60‍ ‍μg L^–1^ H_3_BO_3_, 200‍ ‍μg L^–1^ CoCl_2_·6H_2_O, 20‍ ‍μg L^–1^ CuCl_2_·2H_2_O, 20‍ ‍μg L^–1^ NiCl_2_·6H_2_O, and 40‍ ‍μg L^–1^ Na_2_MoO_4_·H_2_O, pH adjusted to 6.8, and Ormerod agar (2.0% [w/v] agar) (Ormerod *et al.*, 1961), composed of 2.0‍ ‍g‍ ‍L^–1^ sodium malate, 0.1‍ ‍g‍ ‍L^–1^ yeast extract, 0.2‍ ‍g‍ ‍L^–1^ MgSO_4_·7H_2_O, 0.08‍ ‍g‍ ‍L^–1^ CaCl_2_·H_2_O, 0.01‍ ‍g‍ ‍L^–1^ FeSO_4_·7H_2_O, 0.9‍ ‍g‍ ‍L^–1^ K_2_HPO_4_, 0.6‍ ‍g‍ ‍L^–1^ KH_2_PO_4_, 1.25‍ ‍g‍ ‍L^–1^ (NH_4_)_2_SO_4_, 0.02‍ ‍g‍ ‍L^–1^ ethylenediaminetetraacetic acid sodium salt (EDTA-Na), 0.028‍ ‍g‍ ‍L^–1^ H_3_BO_3_, 0.021‍ ‍g‍ ‍L^–1^ MnSO_4_·4H_2_O, 0.075‍ ‍g‍ ‍L^–1^ Na_2_MoO_4_·2H_2_O, 0.0024‍ ‍g‍ ‍L^–1^ ZnSO_4_·2H_2_O, and 0.01‍ ‍g‍ ‍L^–1^ Cu(NO_3_)_2_·3H_2_O, pH adjusted to 7.0. Agricultural wastewater, directly used as a medium component, was sterilized by autoclaving or filtration and was then used to prepare agar (20‍ ‍g‍ ‍L^–1^) or gellan gum (0.8‍ ‍g‍ ‍L^–1^) plates. No cations were added to agricultural wastewater for the preparation of gellan gum plates because wastewater contained a sufficient amount of cations to gelate the plates. Series-diluted agricultural wastewater was then spread on the plates and incubated aerobically at 24°C. Single colonies appearing on plates were selected and re-cultured in 20‍ ‍mL of a rich medium containing 10‍ ‍g‍ ‍L^–1^ peptone, 5‍ ‍g‍ ‍L^–1^ NaCl, and 6‍ ‍g‍ ‍L^–1^ yeast extract at 30°C for 48‍ ‍h with rotary shaking at 168‍ ‍rpm. Cultured bacterial cells were preserved at –80°C as 25% (w/v) glycerol stock before use. Isolated strains were named by alphabetical letters and numbers, with JM, OR, AF, AA, GF, and GA indicating the following isolation media: JCM520 agar, Ormerod agar, filtrated agricultural wastewater agar, autoclaved agricultural waste agar, filtrated agricultural wastewater gellan gum, and autoclaved agricultural wastewater gellan gum, respectively.

### Screening of CGPBs

*Synechococcus elongatus* PCC 7942 purchased from the Pasteur Culture Collection of *Cyanobacteria* (Institute Pasteur, France) was cultivated at 25°C for 7 days in BG11 medium. To select CGPBs, all bacterial strains and *S. elongatus* were cultured using a co-culture medium (Liu *et al.*, 2021). To prepare a seed culture of *S. elongatus*, the cyanobacterial strain was cultivated in a 100-mL Erlenmeyer flask containing 40‍ ‍mL of the co-culture medium at 30°C for 7 days with rotary shaking at 120‍ ‍rpm in a thermostatic shaking incubator (Bioshaker BR-43FL; Taitec) under continuous 115–120‍ ‍μmol m^–2^ s^–1^ photosynthetic photon flex density (PPFD) illumination (white LED irradiation unit, LC-450EXP; Taitec). Isolates from the glycerol stock (50-μL aliquots) were cultured in 1‍ ‍mL of co-culture medium in 96-deep well plates at 30°C for 48‍ ‍h with shaking at 1,200‍ ‍rpm in a Deep Well Maximizer Bioshaker (M·BR-022UP; Taitec). The prepared *S. elongatus* (adjusted as 0.05 of OD_730_ Unit) and bacterial cells (adjusted as 0.01 of OD_600_ Unit) were inoculated into 200‍ ‍μL of the co-culture medium in a 96-well microplate (Violamo Plate; As One). The microplate was incubated at 30°C with shaking at 600‍ ‍rpm using a microplate mini shaker (PSU-2T; Biosan) in a growth chamber (MLR-352; PHCbi) under continuous 115–120‍ ‍μmol m^–2^ s^–1^ PPFD illumination for 6 days, which was shorter than the normal flask cultivation of *S. elongatus*. To evaluate the growth-promoting effects of each bacterial strain, the intensity of chlo­rophyll fluorescence was measured at excitation and emission wavelengths of 488 and 683‍ ‍nm, respectively, as an index of the growth of *S. elongatus* using a fluorescence plate reader (Varioskan LUX; Thermo Fisher Scientific). The fluorescence intensity of monocultured bacterial cells was also measured as the control.

### Taxonomical ana­lysis of CGPBs

The genomic DNAs of CGPB strains were prepared using the alkaline-SDS method, with some modifications (Sambrook *et al.*, 1989). CGPB culture broth (1‍ ‍mL) was centrifuged at 10,000×*g* at 4°C for 1‍ ‍min. Each cell pellet was resuspended in 560‍ ‍μL Tris-EDTA buffer (pH 8.0), and 10‍ ‍μL of 10‍ ‍mg mL^–1^ proteinase K (Wako Pure Chemical), and 30‍ ‍μL of 10% (w/v) SDS was then added. The suspension was incubated at 37°C for 1 h. Genomic DNA was twice extracted with phenol/chloroform/isoamyl alcohol (48:24:1) and purified by isopropanol precipitation. The partial 16S ribosomal RNA (16S rRNA) gene was amplified with the primers 27F (5′-AGRGTTYGATYMTGGCTCAG-3′) and 1492R (5′-RGYTACCTTGTTACGACTT-3′). Amplification was performed on a T-100 thermocycler (Bio-Rad) using the following protocol: initial denaturation at 95°C for 3‍ ‍min, followed by 30 cycles at 95°C for 30‍ ‍s, 55°C for 30‍ ‍s, and 72°C for 2‍ ‍min, with a final elongation at 72°C for 6‍ ‍min. Amplified products were purified using the Wizard SV Gel and PCR Clean-Up system (Promega). Sequences were generated from the purified products with BigDye Terminator Cycle Sequencing kit v3.1 (Thermo Fisher Scientific) and the following primers: 27F (5′-AGRGTTYGATYMTGGCTCAG-3′), 341F (5′-CAATGGRSGVRASYCTGAHS-3′), 909F (5′-AAACTYAAARRAATTGACGG-3′), P699D (5′-YAACGAGCGMRACCC-3′), 1492R (5′-RGYTACCTTGTTACGACTT-3′), P699R (5′-GGGTYKCGCTCGTTR-3′), 518R (5′-ATTACCGCGGCTGCTGG-3′), and 338R (5′-TGCTGCCTCCCGTAGGAGT-3′) (Klindworth *et al.*, 2013). The resulting sequences were analyzed on an ABI Prism 3130 DNA analyzer (Applied Biosystems Hitachi). After a BLAST search ana­lysis against the NCBI GenBank database (http://www.ncbi.nlm.nih.gov/), sequences were aligned in ClustalW v2.0 and subjected to a phylogenetic ana­lysis by the neighbor-joining method (Larkin *et al.*, 2007). A phylogenetic tree was generated using TreeView v1.6.6 (Page, 1996). DNA sequences were deposited in the DDBJ database under accession numbers LC731267–LC731270. To examine morphological characteristics, CGPBs were cultured in AccuDia^TM^ Standard Method Agar (Shimadzu Diagnostics Corporation) at 30°C for 72 h, and were observed under a microscope (Nikon Ts2R, Nikon) with a ×100 magnitude lens using the difference interface method. Gram staining was performed using a common procedure. Microscopic images were obtained by a digital camera system (Ti2; Nikon). Cell sizes were measured by imaging software (NIS-Elements, Nikon).

### Flask cultivation

Inocula of isolated strains and *S. elongatus* were prepared by 100-mL shake flask cultivation in the described rich medium and BG11, respectively. The inoculation ratio and number of days of the CGPB pre-culturation used for flask cultivation were those that exerted the strongest growth-promoting effects on *S. elongatus* in preliminary investigations. CGPB seed cultures were prepared by shake flask cultivation in 100-mL Erlenmeyer flasks containing 40‍ ‍mL of the co-culture medium at 30°C with rotary shaking at 168‍ ‍rpm. Co-cultures, which were conducted using the same culture medium as in the screening experiment, were performed in 100-mL Erlenmeyer flasks containing 40‍ ‍mL of the co-culture medium at 30°C for 7 days with rotary shaking at 120‍ ‍rpm.

Chlorophyll contents were measured as described in a previous study (Broddrick *et al.*, 2016). Following cultivation, 1-mL samples of culture broth were collected and centrifuged at 15,000×*g* at 4°C for 7‍ ‍min. The supernatant was removed, and cell pellets were resuspended in 1‍ ‍mL of chilled methanol. To extract chlo­rophyll from cells, samples were incubated at 4°C for 1‍ ‍h in the absence of light. The absorbance of each sample at 665 and 720‍ ‍nm was measured using a Thermo Scientific Multiskan Sky spectrometer (Thermo Fisher Scientific), with methanol as the blank for calibration purposes. The concentration of chlo­rophyll *a* was calculated using the following equation:

Chl a (μg mL^–1^)=12.9447(A_665_–A_720_)

*S. elongatus* cell numbers and forward scattering and chlo­rophyll fluorescence intensity per cell were measured using a CyFlow Cube 8 flow cytometer (Sysmex) equipped with a 488-nm laser. The voltages of forward scattering (FSC-H), side scattering (SSC-H), and chlo­rophyll fluorescence (FL2-H filter; cut-off of 675‍ ‍nm) were set at 200.0, 275.0, and 675.0 V, respectively. To detect *S. elongatus*, detection gating was set by the fluorescence intensity of chlo­rophyll in flow cytometry software. We herein confirmed that background fluorescence derived from the isolated strains may be ignored in the ana­lysis. The morphological assay of *S. elongatus* using a microscope was performed using the above described methods.

### Indole-3-acetic acid (IAA) and siderophore assays

IAA production was estimated using a colorimetric method based on Salkowski reagent (Ehmann, 1977). Strains were cultivated in IAA production medium containing 10‍ ‍g‍ ‍L^–1^ peptone, 6.0‍ ‍g‍ ‍L^–1^ yeast extract, 1.0‍ ‍g‍ ‍L^–1^ L-tryptophan, and 5.0‍ ‍g‍ ‍L^–1^ NaCl at 30°C for 2 days. After cultivation, the supernatant was isolated by centrifugation at 3,000×*g* at 4°C for 15‍ ‍min. Two hundred microliters of the supernatant, 10‍ ‍μL of 3.5‍ ‍mM phosphate buffer (pH 7.0), and 400‍ ‍μL of Salkowski reagent were mixed on a microplate and incubated for 1‍ ‍h in the dark. Absorbance at 535‍ ‍nm was measured using a microplate reader. IAA concentrations were calculated by a standard curve. *Pseudomonas simiae* OLi (DSM 18861), which was obtained from the German Collection of Microorganism and Cell Cultures GmbH (DSMZ, German), was used as the positive control in the IAA assay. Colorimetric measurements were conducted in triplicate. Siderophore production was analyzed using the chromo azurol S (CAS) blue agar assay (Schwyn and Neilands, 1987). *P. fluorescens* NBRC 14160, which was obtained from the National Biological Resource Center (NBRC), the National Institute of Technology and Evaluation, Japan, was used as a siderophore positive control.

### Whole-cell hydrogenase assay

A hydrogenase assay was performed as described in a previous study (Sebastiampillai *et al.*, 2022). Isolated strains were cultured in modified tryptone-yeast extract-Tris (TYET) including 10‍ ‍g‍ ‍L^–1^ tryptone, 5‍ ‍g‍ ‍L^–1^ yeast extract, 50‍ ‍mM Tris-HCl (pH 7.5), 4‍ ‍g‍ ‍L^–1^ glucose 30‍ ‍mM formate, 1‍ ‍μM sodium selenite, and 1‍ ‍μM sodium molybdate, which had been prepared through filtration with 0.22-μm polyethersulfone membrane. Cells of the isolated strain with 0.1 OD_630_ unit were inoculated into 200‍ ‍μL of TYET broth in a 96-well plastic plate (Violamo Plate; As One Corporation) and then incubated at 30°C for 6 h. After the incubation, OD_630_ was measured using a plate reader (Varioskan LUX; Thermo Fisher Scientific). A developing solution (20‍ ‍μL), including 10‍ ‍mg mL^–1^ benzyl viologen and 250‍ ‍mM sodium formate in 20‍ ‍mM Tris-HCl buffer (pH 7.5), was added to each well. Changes in A_630_ were monitored by the plate reader every 30‍ ‍s for 5‍ ‍min. Increases in A_630_ per OD_630_ for 1‍ ‍min were calculated from the time course.

## Results

### Screening of CGPBs

**Fig. 1.**
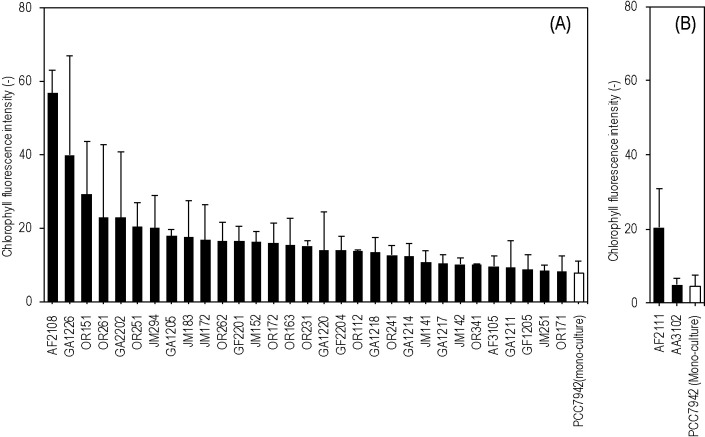
The co-culture combination with *Synechococcus elongatus* PCC 7942 in the co-culture screening experiment in which the chlo­rophyll fluorescence intensity of *S. elongatus* PCC 7942 was more than 1.0-fold higher than that in the mono-culture of *S. elongatus* PCC 7942 on day 6. Bar graphs (A) and (B) show two batches of the screening experiment conducted in the present study. Error bars indicate standard deviations (*n*=3).

In the present study, we isolated and screened 144 potential CGPB strains for their effects on the growth of *S. elongatus*. Due to equipment limitations, the screening experiment was conducted in two batches. In the first CGPB screening, 33 strains increased chlo­rophyll fluorescence when co-cultured with *S. elongatus* (Fig. 1). Strains AF2108, GA1226, AF2111, and OR151 enhanced the growth of co-cultured *S. elongatus* by 7.5-, 5.3-, 4.7-, and 3.9-fold, respectively, from that in the monoculture. These strains were selected for subsequent experiments.

### Taxonomical ana­lysis

**Fig. 2.**
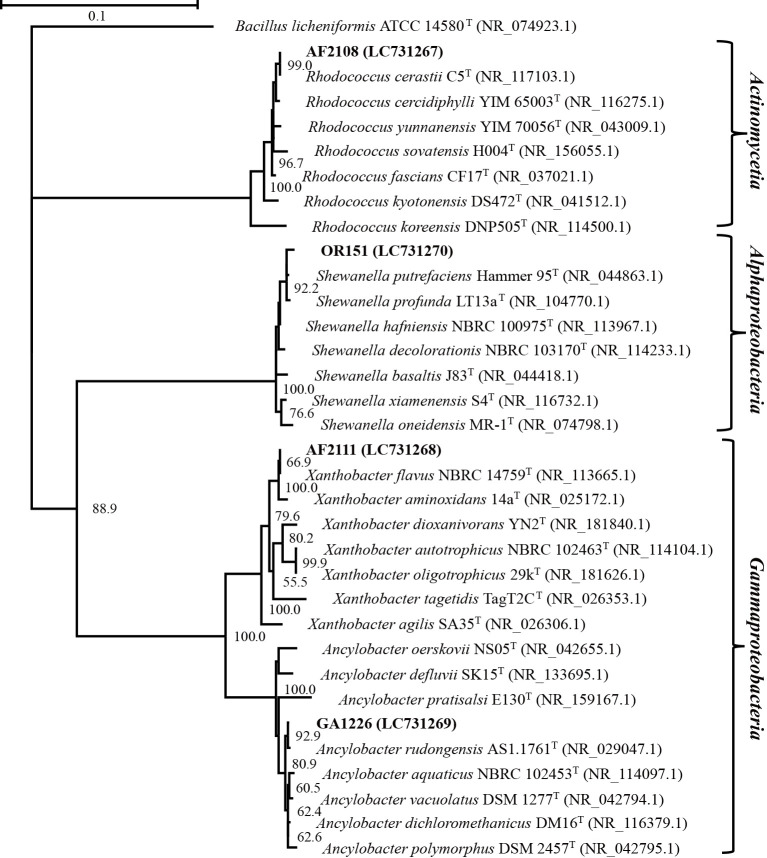
16S rRNA-based phylogenetic reconstruction of four aerobic heterotrophic bacterial strains used in the present study. *Bacillus licheniformis* ATCC 14580^T^ was set as the outgroup. Bootstrap value ≥50%.

According to the phylogenetic ana­lysis of their 16S rRNA gene sequences, the four isolated CGPB strains belonged to three classes: *Actinomycetia*, *Alphaproteobacteria*, and *Gammaproteobacteria* (Fig. 2). Strain AF2108 was closely related (99.95% sequence similarity) to the Gram-positive actinomycete *Rhodococcus cerastii* C5 (Kämpfer *et al.*, 2013). Morphological observations (Fig. S1) showed that AF2108 was a Gram-positive, non-motile, and short-rod (length of 1.09–3.48‍ ‍μm and width of 0.39–0.98) strain. Colonies were round, convex, and yellowish orange in color. These morphological characteristics corresponded to those of the type strain *R. cerastii* C5 (Kämpfer *et al.*, 2013). Therefore, strain AF2108 was identified as *Rhodococcus* sp., which was closely related to *R. cerastii*. In the phylogenetic tree (Fig. 2), strains GA1226 and AF2111 belonged to the *Xanthobacteraceae* family of *Gammaproteobacteria* and were closely related to *Ancylobacter rudongensis* AS1.1761^T^ and *Xanthobacter flavus* NBRC 14759^T^, with 99.56 and 100.0% sequence similarities, respectively. GA1226 was a Gram-negative, non-motile, curved-rod (length of 1.55–3.65‍ ‍μm and width of 0.45–0.94‍ ‍μm) strain (Fig. S1). Colonies were white, round, convex, and opaque, which corresponded to the type strain *A. rudongensis* (Xin *et al.*, 2004). AF2111 was a Gram-negative, rod-shape (length of 1.00–4.56‍ ‍μm and width of 0.58–1.02‍ ‍μm) strain. Colonies were yellow, round, entire, convex, and opaque. These morphological characteristics corresponded to those of the type strain *X. flavus* (Malik and Claus, 1979). Therefore, strains GA1226 and AF2111 were assigned as *Ancylobacter* sp. and *Xanthobacter* sp., respectively. The phylogenetic ana­lysis placed strain OR151 within the genus *Shewanella*. OR151 was a Gram-negative, motile, rod-shaped (length of 1.04–2.43‍ ‍μm and width of 0.39–0.97‍ ‍μm) strain. Colonies were beige, circular, and convex. In the phylogenetic tree, strain OR151 was the most closely related to *Shewanella putrefaciens* and *Shewanella profunda*; however, the strain was not assigned to either species based on the partial 16S rRNA sequence and, thus, was designated as *Shewanella* sp. OR151. According to the phylogenetic ana­lysis, the four CGPB strains were widely distributed across bacterial phyla.

### Evaluation of cyanobacterial growth promotion in flask-scale cultures

**Fig. 3.**
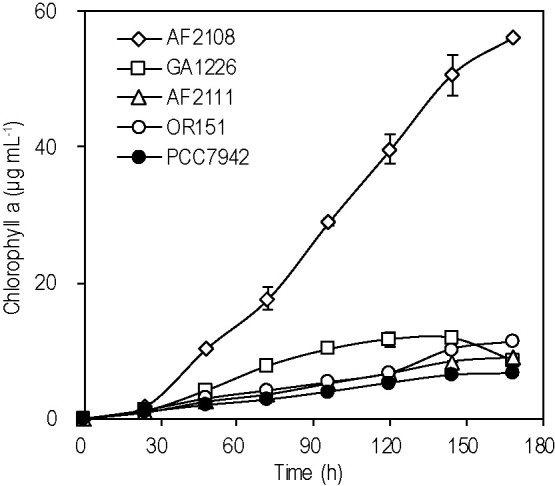
The growth of *Synechococcus elongatus* co-cultured with and without CGPB strains (AF2108, GA1226, AF2111, and OR151) in the flask scale co-culture experiment. The growth of *S. elongatus* was monitored by measuring the content of chlo­rophyll a. Error bars indicate standard deviations (*n*=3).

After optimizing seed culture periods and CGPB inoculation rates in multi-well plate cultures (Fig. S2), we performed flask co-cultures to confirm the growth-promoting activity of the CGPB strains in more detail. The seed culture periods and inoculation rates of *Rhodococcus* sp. AF2108, *Ancylobacter* sp. GA1226, *Xanthobacter* sp. AF2111, and *Shewanella* sp. OR151 were set to 1 day and OD_600_=0.02, 1 day and OD_600_=0.04, 2 days and OD_600_=0.02, and 2 days and OD_600_=0.02, respectively. As shown in Fig. 3, we monitored the content of chlo­rophyll *a* over time as a cyanobacterial growth index during flask-based cyanobacteria–CGPB co-cultivation and cyanobacterial monocultivation. When *Rhodococcus* sp. AF2108 was co-cultured with *S. elongatus*, the content of chlo­rophyll *a* derived from *S. elongatus* was 8.5-fold higher after 168‍ ‍h than that of the monocultured cyanobacterium. At each corresponding time point, the other CGPBs were found to exert weaker growth-promoting effects. The highest fold change in growth observed in the co-culture of *Ancylobacter* sp. GA1226 with *S. elongatus* from that in the monoculture with the cyanobacterium alone, 2.8-fold, was recorded after 72 h. When co-cultured with *S. elongatus*, *Xanthobacter* sp. AF2111 and *Shewanella* sp. OR151 increased cyanobacterial growth by 1.3- and 1.7-fold, respectively, after 168 h. However, the growth-promoting effects of *Xanthobacter* sp. AF2111 and *Shewanella* sp. OR151 co-cultured with *S. elongatus* in flasks were weaker than those obtained using multi-well microplates. These differences in cyanobacterial growth promotion were caused by variations in physical conditions (other than temperature), such as the aeration level (agitation rate) and light intensity, between the two experiments due to differences in vessel sizes. Regardless of these differences between culture scales, *Rhodococcus* sp. AF2108 exhibited highly stable cyanobacterial growth-promoting activity. According to flow cytometric data, the numbers of *S. elongatus* cells counted in co-cultures with *Rhodococcus* sp. AF2108, *Ancylobacter* sp. GA1226, and *Shewanella* sp. OR151 were 3.9-, 2.0-, and 1.5-fold higher than that in the corresponding monoculture (Table 1). In contrast, co-culturing *S. elongatus* with *Xanthobacter* sp. AF2111 did not significantly increase the number of cyanobacterial cells counted. The intensities of forward and side scattering as the index of cell size were measured in the present study. Even though the intensity of side scattering was 0.9-fold weaker in both co-cultures with *Rhodococcus* sp. AF2108 and *Shewanella* sp. OR151, forward scattering was 1.5- and 1.7-fold larger, respectively, in co-cultures with the aforementioned strains than that of monocultured cyanobacteria. On the other hand, no significant differences were observed between co-cultures and the monoculture in the case of *Ancylobacter* sp. GA1226 and *Xanthobacter* sp. AF2111 (Table 1). To confirm cell morphology during co-cultures, microscope observations were performed (Table S1). The cell width of cyanobacterial cells markedly increased in cases of *Rhodococcus* sp. AF2108 and *Shewanella* sp. OR151. The observed cell area in cases of *Rhodococcus* sp. AF2108 and *Shewanella* sp. OR151 also corresponded to the results of forward scattering by flow cytometry. According to microscopic observations, *Ancylobacter* sp. GA1226 and *Xanthobacter* sp. AF2111 did not appear to affect cyanobacterial cell sizes. Cell size depends on the basic processes of cell physiology, and changes in cell size have a profound impact on metabolic flux, biosynthetic capacity, and nutrient exchange (Marshall *et al.*, 2012). In a previous study, cell enlargement was noted following supplementation with metal ions (Nishino *et al.*, 2018). We also measured the chlo­rophyll fluorescence intensity per cell of *S. elongatus* under each cultivation condition (Table 1). After co-culturing with *Rhodococcus* sp. AF2108 or *Shewanella* sp. OR151, chlo­rophyll fluorescence intensities per cell were 2.0- and 1.3-fold higher, respectively, than that of the cyanobacterium in the monoculture; therefore, co-culturing with *Ancylobacter* sp. GA1226 or *Xanthobacter* sp. AF2111 did not significantly affect chlo­rophyll fluorescence intensity per cell. These results revealed that co-cultivations with different partner strains led to not only in differences in the total amount of chlo­rophyll *a*, but also the total number of cells, cell size, and chlo­rophyll fluorescence intensity per cell of *S. elongatus*.

### Siderophore production, IAA production, and whole-cell hydrogenase activity

To estimate the cyanobacterial growth promoting factors of the CGPBs obtained, siderophore production, IAA production, and whole-cell hydrogenase activity were measured (Table S2). Siderophore production by *Rhodococcus* sp. AF2108 and *Shewanella* sp. OR151 was positive in the CAS blue agar assay, with *P. fluorescens* as the positive control, whereas that by *Ancylobacter* sp. GA1226 and *Xanthobacter* sp. AF2111 was only weakly positive. All CGPB strains produced IAA; however, the amount of IAA was less than 18% of the positive control, *P. simiae*. Whole-cell hydrogenase activity was observed for *Shewanella* sp. OR151, but not for the other strains.

## Discussion

In the present study, CGPBs were successfully isolated and efficiently enhanced the growth of the cyanobacterium *S. elongatus*. Table 2 summarizes the results of various studies on the enhancing effects of co-cultures on the growth of cyanobacteria. Isolates of *Rhodococcus* sp., *Ancylobacter* sp., *Xanthobacter* sp., and *Shewanella* sp. were described as CGPBs in the present study. In a previous study, *Pseudomonas* sp. GM41, which was identified as the most effective CGPB analyzed (Abed, 2010), induced an 8-fold increase in the cyanobacterial content of chlo­rophyll during a 20-day co-cultivation with the cyanobacterium *Synechocystis* PCC6803 in the presence of hexadecane. In the present study, *Rhodococcus* sp. AF2108 increased the content of chlo­rophyll *a* in *S. elongatus* by 8.5-fold during a 7-day co-cultivation (Fig. 3). This result indicates that *Rhodococcus* sp. AF2108 exerted stronger growth-promoting effects over a shorter period than *Pseudomonas* sp. GM41; therefore, to the best of our knowledge, *Rhodococcus* sp. AF2108 is the most effective CGPB described to date. *Synechococcus* is a promising host for producing biomass and metabolites, such as those generated by photosynthesis from atmospheric carbon dioxide via synthetic metabolic pathways, and may be genetically modified (Oliver and Atsumi, 2015). Nevertheless, the low proliferation rate of *Synechococcus* limits production efficiency. Difficulties are associated with enhancing the growth rate through improvements in the flux of total metabolic pathways via metabolic engineering. Even though the mechanisms responsible for the observed growth promotion are presently unknown, the CGPBs isolated in the present study may contribute to a new *Synechococcus* growth improvement strategy. A CGPB–*Synechococcus* co-culture system may be applied to various bioprocesses using autotrophic *Synechococcus* metabolic pathways. We noted that co-culturing with *Rhodococcus* sp. AF2108 increased cyanobacterial growth, as assessed by the content of chlo­rophyll and cell number indexes, by 8.5-fold (Fig. 3) and 3.9-fold (Table 1), respectively, over that in the cyanobacterial monocultivation.

In the present study, we did not examine the mechanisms underlying cyanobacterial growth enhancements. Based on previous findings on plant growth-promoting bacteria (PGPBs) and microalgae growth-promoting bacteria (MGPBs), four hypotheses have been proposed to explain how CGPBs enhance cyanobacterial growth: (1) by exchanging nutrients between CGPBs and cyanobacteria, (2) by enhancing nitrogen fixation via CGPBs, (3) by signaling via phytohormones or phytohormone-like factors, and (4) by enhancing the uptake of insoluble materials by siderophores.

Cyanobacteria exude dissolved organic matter, which becomes available for bacteria. In return, bacteria remineralize sulfur, nitrogen, and phosphorus to support the further growth of cyanobacteria (Buchan *et al.*, 2014). In specific interactions, bacteria supply vitamin B group derivatives as organic cofactors or produce siderophores to bind iron; siderophores increase the bioavailability of iron for cyanobacteria, while cyanobacteria, in return, provide dissolved organic carbon for bacteria (Croft *et al.*, 2005; Amin *et al.*, 2012). In a previous study, *Rhodococcus* sp. increased the biomass of *Chlamydomonas reinhardtii* cc124 by 46% over that of the monocultured microalgal strain (Lakatos *et al.*, 2014). In addition, *Rhodococcus* sp. promoted microalgal growth and hydrogen production during co-cultivation, which indicated that oxygen elimination is the most crucial factor for algal hydrogen production and that efficient bacterial respiration is essential for the activation of algal Fe-hydrogenase. At the same time, increased carbon dioxide emissions from bacteria may enhance carbon fixation by microalgae. In the present study, co-culturing with *Rhodococcus* sp. AF2108 resulted in cyanobacterial growth that was 8.5-fold (Fig. 3) and 3.9-fold (Table 1) higher, according to the content of chlo­rophyll and cell number indexes, respectively, than that of cyanobacteria in a monoculture. These fold changes in growth were larger than that in a previous study, which reported a 1.46-fold increase in *C. reinhardtii* cc124 biomass (Lakatos *et al.*, 2014). Although the cyanobacterial growth-promoting mechanism of *Rhodococcus* sp. AF2108 is unknown, the marked increase observed in the growth of *S. elongatus* cannot be solely explained—unlike in the previous study (Lakatos *et al.*, 2014)—by enhanced proliferation and respiration due to decreases and increases in the levels of dissolved oxygen and carbon dioxide, respectively.

*Xanthobactor autotrophicus* is a bacterial species that fixes nitrogen by simultaneously expressing hydrogenase and nitrogenase. The genus *Xanthobacter* is also exploited for sustainable ammonia and biofertilizer production (Liu *et al.*, 2017). However, *Xanthobacter* sp. AF2111 did not exhibit whole-cell hydrogenase activity (Table S2). Based on the present results, nitrogen fixation by *Xanthobacter* sp. AF2111 did not appear to promote cyanobacterial growth.

In a previous study, plant-associated *Rhodococcus qingshengii* RL1, in the *Actinomycetia* clade in the phylogenetic tree (Fig. 2), exhibited the ability to produce the phytohormone, IAA (Kuhl *et al.*, 2021). In the case of microalgae, extracellular IAA has been reported to enhance algal growth (Dao *et al.*, 2018); however, it currently remains unclear whether IAA enhances the growth of *Synechococcus*. In a previous study in which cyanobacteria were exposed to chromium, exogenous supplementation with IAA and kinetin (a cytokinin-like synthetic phytohormone) attenuated the effects of chromium toxicity by decreasing chromium uptake (Tiwari *et al.*, 2020). In the present study, all CGPBs produced a small amount of IAA from tryptophan (Table S2). Therefore, IAA produced by the strains during the co-culture may have stimulated cyanobacterial growth. However, it may not the sole factor promoting cyanobacterial growth because the ability to produce IAA was markedly weaker than that of the positive control species, *P. simiae*.

Members of the genus *Shewanella* decrease toxicity in the environment by reducing various electron acceptors (Dikow, 2011). In a previous study, a species of *Shewanella* rapidly protected *Synechococcus* sp. PCC7002 from Fe^2+^ toxicity before major oxidation induced cellular damage (Szeinbaum *et al.*, 2021). Although iron plays a key role in cyanobacterial physiology, an excess of free intracellular iron is extremely harmful because it catalyzes the formation of reactive oxygen species (ROS) through Fenton reactions, leading to oxidative stress (Sauer *et al.*, 2001). Therefore iron uptake and metabolism need to be tightly regulated in order to ensure a supply that maintains intracellular concentrations within non-toxic levels (González *et al.*, 2012). In a previous study, *S. putrefaciens* W3-18-1 increased iron availability and provided more facile, energy-efficient mechanisms for iron acquisition by *Synechococcus* sp. PCC7002, a conclusion inferred from the broad and consistent decrease in the mRNA levels of Fe-regulated *Synechococcus* sp. PCC7002 genes during the co-cultivation (Beliaev *et al.*, 2014). These findings showed that *Shewanella* may have expanded the upper tolerable Fe^2+^ concentration and increased the uptake of iron for the cyanobacterium, and also suggest that the growth of *S. elongatus* was promoted by siderophores produced by *Shewanella* sp. OR151. Although cyanobacteria produce siderophores, such as schizokinen and synechobactin A (Årstøl and Hohmann-Marriott, 2019), they still require high levels of iron for photosynthesis (Sunda and Huntsman, 2015). By chelating insoluble ferric ions, siderophores help increase iron uptake by cyanobacteria (Neilands, 1995). Siderophores, such as putrebactin, bisucaberin, and avaroferrin, have been identified in the genus *Shewanella* (Liu *et al.*, 2022). In the present study, the CAS blue agar assay showed that siderophore production by *Rhodococcus* sp. AF2108 and *Shewanella* sp. OR151 was positive (Table S2), whereas that by the other strains was only weakly positive. Based on these results, siderophores produced by the two strains may have promoted cyanobacterial growth during the co-culture. Furthermore, the siderophore-positive strains, *Rhodococcus* sp. AF2108 and *Shewanella* sp. OR151, increased the cell size of *S. elongatus*. Although siderophores appeared to affect cyanobacterial cell size, further evidence is needed.

## Conclusion

In the present study, four novel CGPB strains, *Rhodococcus* sp. AF2108, *Ancylobacter* sp. GA1226, *Xanthobacter* sp. AF2111, and *Shewanella* sp. OR151, were isolated and exerted growth-promoting effects on *S. elongatus*; these CGPBs increased the content of chlo­rophyll *a* in *S. elongatus* by 8.5-, 2.8-, 1.3-, and 1.7-fold, respectively. According to flow cytometry, the four CGPBs also increased the cell number, forward scattering intensity, and chlo­rophyll fluorescence intensity per cell of *S. elongatus* to varying extents. *Rhodococcus* sp. AF2108 exerted faster and stronger growth-promoting effects than bacteria in previous studies, which indicates that *Rhodococcus* sp. AF2108 is the most effective CGPB strain identified to date. These four newly isolated CGPBs will serve as important components of a new *Synechococcus* growth improvement strategy. The present results indicate that IAA and siderophores stimulate cyanobacterial growth. These CGPBs may be applied to various bioprocesses that rely on autotrophic *Synechococcus* metabolic pathways.

## Citation

Tan, P. Y., Kato, Y., and Konishi, M. (2024) A Novel Strain of the Cyanobacterial Growth-promoting Bacterium, *Rhodococcus* sp. AF2108, Enhances the Growth of *Synechococcus elongatus*. *Microbes Environ ***39**: ME24050.

https://doi.org/10.1264/jsme2.ME24050

## Supplementary Material

Supplementary Material

## Figures and Tables

**Table 1.** Flow cytometry specifically analyzed *Synechococcus elongatus* on day 7 after the co-culture with CGPBs and the monoculture.

	Number of cells counted (×10^8^ mL^–1^)	Forward scatter intensity per cell (×10^2^)	Side scatter intensity per cell (×10)	Chlorophyll fluorescence intensity cell^–1^ (×10^2^)
Co-culture with AF2108	17.8±0.8*	13.9±0.4*	4.95±0.37*	1.94±0.18*
Co-culture with GA1226	9.3±0.3*	8.1±0.0*	5.48±0.95	1.21±0.10
Co-culture with AF2111	5.8±0.6	9.6±0.4	6.07±0.50	1.46±0.25
Co-culture with OR151	6.7±0.3*	14.9±0.3*	4.96±0.12*	1.24±0.10*
Monoculture of *S. elongatus*	4.6±0.1	9.1±0.1	5.64±0.06	0.98±0.09

Note: Values are means±standard deviations (*n*=3). Asterisks indicate significant differences (*P*<0.05) from *S. elongatus* (monoculture). The significance of differences was calculated by the Tukey-Kramer method.

**Table 2.** Effects of co-culturing cyanobacteria with bacteria or microalgae reported from various studies.

Co-culture		Culture time (day)	Product	Fold change	Ref.
*Cyanobacteria*	Co-culture microbes				
*Synechococcus elongatus* PCC 7942	*Rhodococcus* sp. AF2108	7	Biomass (Chlorophyll *a*)	8.5	This study
	*Ancylobacter* sp. GA1226			2.8	
	*Xanthobacter* sp. AF2111			1.3	
	*Shewanella* sp. OR151			1.7	
*Synechococcus elongatus* PCC 7942	*Rhodotorula glutinis* ATCC 204091	30	Lipid yield	1.4–1.6	Li *et al.*, 2017
*Synechocystis* sp. PCC6803	*Pseudomonas* sp. GM41	20	Biomass (Chlorophyll *a*)	8	Abed, 2010
*Synechocystis salina* LEGE 06079	*Chlorella vulgaris* CCAP 211/11B	7	Biomass (dry cell weight)	1.62	Gonçalves *et al.*, 2016
	*Pseudokirchneriella subcapitata* CCAP 278/4			1.07	
	*Microcystis aeruginosa* LEGE 9134			1.40	
*Spirulina platensis* UTEX 1926	*Rhodotorula glutinis* 2.541	5	Lipid production	3.92	Xue *et al.*, 2010

## References

[B1] Abed, R.M.M. (2010) Interaction between cyanobacteria and aerobic heterotrophic bacteria in the degradation of hydrocarbons. Int Biodeterior Biodegrad 64: 58–64.

[B2] Amin, S.A., Green, D.H., Gärdes, A., Romano, A., Trimble, L., and Carrano, C.J. (2012) Siderophore-mediated iron uptake in two clades of *Marinobacter* spp. associated with phytoplankton: The role of light. BioMetals 25: 181–192. 21947474 10.1007/s10534-011-9495-5

[B3] Årstøl, E., and Hohmann-Marriott, M.F. (2019) Cyanobacterial siderophores—physiology, structure, biosynthesis, and applications. Mar Drugs 17: 281. 31083354 10.3390/md17050281 PMC6562677

[B4] Beliaev, A.S., Romine, M.F., Serres, M., Bernstein, H.C., Linggi, B.E., Markillie, L.M., et al. (2014) Inference of interactions in cyanobacterial–heterotrophic co-cultures via transcriptome sequencing. ISME J 8: Article 11. 10.1038/ismej.2014.69 PMC4992078 24781900

[B5] Biebl, H., and Pfennig, N. (1981) Isolation of members of the family *Rhodospirillaceae*. In *The Prokaryotes: A Handbook on Habitats, Isolation, and Identification of Bacteria*. Starr, M.P., Stolp, H., Trüper, H.G., Balows, A., and Schlegel, H.G. (eds). Heidelberg: Springer, pp. 267–273.

[B6] Broddrick, J.T., Rubin, B.E., Welkie, D.G., Du, N., Mih, N., Diamond, S., et al. (2016) Unique attributes of cyanobacterial metabolism revealed by improved genome-scale metabolic modeling and essential gene ana­lysis. Proc Natl Acad Sci U S A 113: E8344–E8353. 27911809 10.1073/pnas.1613446113 PMC5187688

[B7] Buchan, A., LeCleir, G., Gulvik, C., and González, J. (2014) Master recyclers: Features and functions of bacteria associated with phytoplankton blooms. Nat Rev Microbiol 12: 686–698. 25134618 10.1038/nrmicro3326

[B8] Croft, M., Lawrence, A., Deery, E., Warren, M., and Smith, A. (2005) Algae acquire vitamin B12 through a symbiotic relationship with bacteria. Nature 438: 90–93. 16267554 10.1038/nature04056

[B9] Dao, G.H., Wu, G.X., Wang, X.X., Zhang, T.Y., Zhan, X.M., and Hu, H.Y. (2018) Enhanced microalgae growth through stimulated secretion of indole acetic acid by symbiotic bacteria. Algal Res 33: 345–351.

[B10] Dikow, R.B. (2011) Genome-level homology and phylogeny of *Shewanella* (Gammaproteobacteria: Lteromonadales: Shewanellaceae). BMC Genomics 12: 237. 21569439 10.1186/1471-2164-12-237 PMC3107185

[B11] Ehmann, A. (1977) The Van Urk-Salkowski reagent a sensitive and specific chromatogenic reagent for silica gel thin-layer chromatographic detection and identification of indol derivatives. J Chromatogr 132: 267–276. 188858 10.1016/s0021-9673(00)89300-0

[B12] Gonçalves, A.L., Pires, J.C.M., and Simões, M. (2016) Biotechnological potential of Synechocystis salina co-cultures with selected microalgae and cyanobacteria: Nutrients removal, biomass and lipid production. Bioresour Technol 200: 279–286. 26496217 10.1016/j.biortech.2015.10.023

[B13] González, A., Bes, M.T., Valladares, A., Peleato, M.L., and Fillat, M.F. (2012) FurA is the master regulator of iron homeostasis and modulates the expression of tetrapyrrole biosynthesis genes in *Anabaena* sp. PCC 7120. Environ Microbiol 14: 3175–3187. 23066898 10.1111/j.1462-2920.2012.02897.x

[B14] Hirokawa, Y., Suzuki, I., and Hanai, T. (2015) Optimization of isopropanol production by engineered cyanobacteria with a synthetic metabolic pathway. J Biosci Bioeng 119: 585–590. 25454065 10.1016/j.jbiosc.2014.10.005

[B15] Kämpfer, P., Wellner, S., Lohse, K., Lodders, N., and Martin, K. (2013) *Rhodococcus cerastii* sp. and *Rhodococcus trifolii* sp. nov. two novel species isolated from leaf surfaces. Int. J Syst Evol Microbiol 63: 1024–1029. 10.1099/ijs.0.044958-0 22685110

[B16] Kato, Y., and Konishi, M. (2024) A mature liquid fertilizer derived from cattle urine promotes *Arabidopsis thaliana* growth via hormone-like responses. Biosci Biotechnol Biochem 88: 1007–1018. 38849314 10.1093/bbb/zbae080

[B17] Klindworth, A., Pruesse, E., Schweer, T., Peplies, J., Quast, C., Horn, M., and Glöckner, F.O. (2013) Evaluation of general 16S ribosomal RNA gene PCR primers for classical and next-generation sequencing-based diversity studies. Nucleic Acids Res 41: 1–11. 22933715 10.1093/nar/gks808 PMC3592464

[B18] Kuhl, T., Chowdhury, S.P., Uhl, J., and Rothballer, M. (2021) Genome-based characterization of plant-associated *Rhodococcus qingshengii* RL1 reveals stress tolerance and plant–microbe interaction traits. Front Microbiol 12: 708605. 34489897 10.3389/fmicb.2021.708605 PMC8416521

[B19] Lakatos, G., Deák, Z., Vass, I., Rétfalvi, T., Rozgonyi, S., Rákhely, G., et al. (2014) Bacterial symbionts enhance photo-fermentative hydrogen evolution of *Chlamydomonas* algae. Green Chem 16: 4716–4727.

[B20] Larkin, M.A., Blackshields, G., Brown, N.P., Chenna, R., McGettigan, P.A., McWilliam, H., et al. (2007) Clustal W and Clustal X version 2.0. Bioinformatics 23: 2947–2948. 17846036 10.1093/bioinformatics/btm404

[B21] Li, T., Li, C.T., Butler, K., Hays, S.G., Guarnieri, M.T., Oyler, G.A., and Betenbaugh, M.J. (2017) Mimicking lichens: Incorporation of yeast strains together with sucrose-secreting cyanobacteria improves survival, growth, ROS removal, and lipid production in a stable mutualistic co-culture production platform. Biotechnol Biofuels 10: 1–11. 28344645 10.1186/s13068-017-0736-x PMC5360037

[B22] Liang, F., Englund, E., Lindberg, P., and Lindblad, P. (2018) Engineered cyanobacteria with enhanced growth show increased ethanol production and higher biofuel to biomass ratio. Metab Eng 46: 51–59. 29477858 10.1016/j.ymben.2018.02.006

[B23] Liu, C., Sakimoto, K.K., Colón, B.C., Silver, P.A., and Nocera, D.G. (2017) Ambient nitrogen reduction cycle using a hybrid inorganic–biological system. Proc Natl Acad Sci U S A 114: 6450–6455. 28588143 10.1073/pnas.1706371114 PMC5488957

[B24] Liu, H., Cao, Y., Guo, J., Xu, X., Long, Q., Song, L., and Xian, M. (2021) Study on the isoprene-producing co-culture system of *Synechococcus elongates*–*Escherichia coli* through omics ana­lysis. Microb Cell Fact 20: 6. 33413404 10.1186/s12934-020-01498-8 PMC7791884

[B25] Liu, L., Wang, W., Wu, S., and Gao, H. (2022) Recent Advances in the Siderophore Biology of Shewanella. Front Microbiol 13: 823758. 35250939 10.3389/fmicb.2022.823758 PMC8891985

[B26] Malik, K.A., and Claus, D. (1979) *Xanthobacter flavus*, a new species of nitrogen-fixing hydrogen bacteria. Int J Syst Evol Microbiol 29: 283–287.

[B27] Marshall, W.F., Young, K.D., Swaffer, M., Wood, E., Nurse, P., Kimura, A., et al. (2012) What determines cell size? BMC Biol 10: 101. 23241366 10.1186/1741-7007-10-101 PMC3522064

[B28] Neilands, J.B. (1995) Siderophores: Structure and function of microbial iron transport compounds. J Biol Chem 270: 26723–26726. 7592901 10.1074/jbc.270.45.26723

[B29] Nishino, K., Morita, Y., Takahashi, S., Okumura, M., Shiratani, S., Umemura, K., et al. (2018) Enlargement of *Deinococcus grandis* spheroplasts requires Mg^2+^ or Ca^2+^. Microbiology 164: 1361–1371. 30222092 10.1099/mic.0.000716

[B30] Oliver, J.W.K., and Atsumi, S. (2015) A carbon sink pathway increases carbon productivity in cyanobacteria. Metab Eng 29: 106–112. 25777135 10.1016/j.ymben.2015.03.006

[B31] Ormerod, J.G., Ormerod, K.S., and Gest, H. (1961) Light-dependent utilization of organic compounds and photoproduction of mole­cular hydrogen by photosynthetic bacteria; relationships with nitrogen metabolism. Arch Biochem Biophys 94: 449–463. 13731247 10.1016/0003-9861(61)90073-x

[B32] Page, R.D. (1996) TreeView: An application to display phylogenetic trees on personal computers. CABIOS Comput Appl Biosci 12: 357–358. 8902363 10.1093/bioinformatics/12.4.357

[B33] Ruffing, A.M. (2014) Improved free fatty acid production in Cyanobacteria with *Synechococcus* sp. PCC 7002 as host. Front Bioeng Biotechnol 2: 17. 25152890 10.3389/fbioe.2014.00017 PMC4126656

[B34] Sambrook, J., Fritsch, E.F., and Maniatis, T. (1989) *Molecular cloning: A Laboratory Manual.* Cold Spring Harbor, NY: Cold Spring Harbor Laboratory Press.

[B35] Sauer, J., Schreiber, U., Schmid, R., Völker, U., and Forchhammer, K. (2001) Nitrogen starvation-induced chlorosis in *Synechococcus* PCC 7942. Low-level photosynthesis as a mechanism of long-term survival. Plant Physiol 126: 233–243. 11351086 10.1104/pp.126.1.233 PMC102297

[B36] Schwyn, B., and Neilands, J.B. (1987) Universal chemical assay for the detection and determination of siderophore. Anal Biochem 160: 47–56. 2952030 10.1016/0003-2697(87)90612-9

[B37] Sebastiampillai, S., Lacasse, M.J., McCusker, S., Campbell, T., Nitz, M., and Zamble, D.B. (2022) Using a high-throughput, whole-cell hydrogenase assay to identify potential small molecule inhibitors of [NiFe]-hydrogenase. Metallomics 14: mfac073. 36190308 10.1093/mtomcs/mfac073

[B38] Singh, J., and Dhar, D.W. (2019) Overview of carbon capture technology: Microalgal biorefinery concept and state-of-the-art. Front Mar Sci 6: 29.

[B39] Sunda, W.G., and Huntsman, S.A. (2015) High iron requirement for growth, photosynthesis, and low-light acclimation in the coastal cyanobacterium *Synechococcus bacillaris*. Front Microbiol 6: 561. 26150804 10.3389/fmicb.2015.00561 PMC4471429

[B40] Szeinbaum, N., Toporek, Y., Reinhard, C.T., and Glass, J.B. (2021) Microbial helpers allow cyanobacteria to thrive in ferruginous waters. Geobiology 19: 510–520. 33871172 10.1111/gbi.12443 PMC8349797

[B41] Tiwari, S., Patel, A., and Prasad, S.M. (2020) Phytohormone up-regulates the biochemical constituent, exopolysaccharide and nitrogen metabolism in paddy-field cyanobacteria exposed to chromium stress. BMC Microbiol 20: 206. 32660415 10.1186/s12866-020-01799-3 PMC7359020

[B42] Wu, L., Quan, L., Deng, Z., Vadiveloo, A., Cheng, Y., Yang, L., et al. (2023) Performance of a biocrust cyanobacteria-indigenous bacteria (BCIB) co-culture system for nutrient capture and transfer in municipal wastewater. Sci Total Environ 888: 164236. 37201839 10.1016/j.scitotenv.2023.164236

[B43] Xin, Y.H., Xhou, Y.G., Zhou, H.L., and Chen, X. (2004) *Ancylobacter rudongensis* sp. nov., isolated from roots of *Spartina anglica*. Int J Syst Evol Microbiol 54: 385–388. 15023948 10.1099/ijs.0.02466-0

[B44] Xue, F., Miao, J., Zhang, X., and Tan, T. (2010) A new strategy for lipid production by mix cultivation of *Spirulina platensis* and *Rhodotorula glutinis*. Appl Biochem Biotechnol 160: 498–503. 18931954 10.1007/s12010-008-8376-z

