# Supplementary material for: A Novel Strain of the Cyanobacterial Growth-promoting Bacterium, Rhodococcus sp. AF2108, Enhances the Growth of Synechococcus elongatus
Source: Microbes Environ. 2024 Dec 28;39(4):ME24050. doi: 10.1264/jsme2.ME24050 (PMC11821763; doi:10.1264/jsme2.ME24050)
Supplement: Supplementary file 1 — Supplementary Material [file 39_24050_s1.pdf]

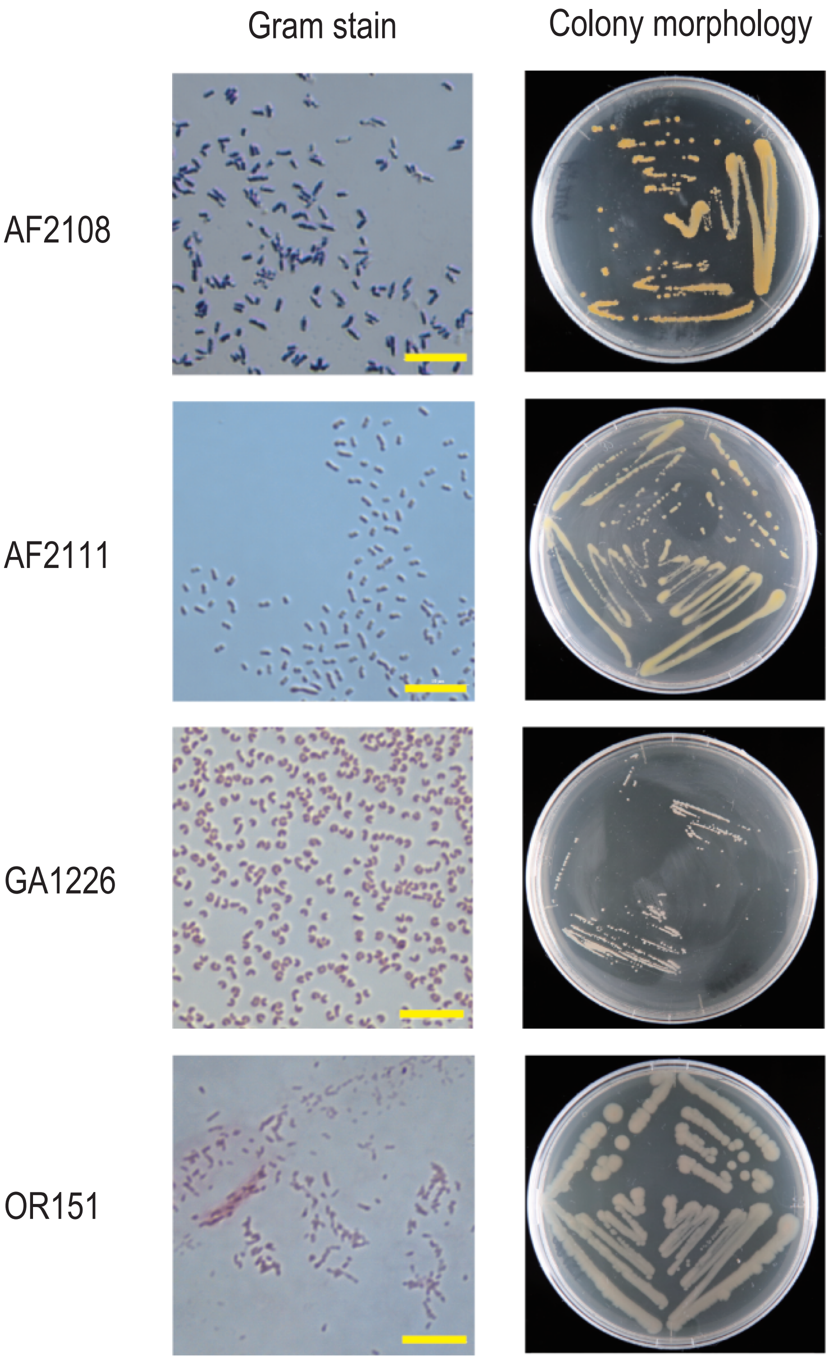

Fig. S1. Morphological data of the isolated strains. Scale bars indicate 10 μm length.

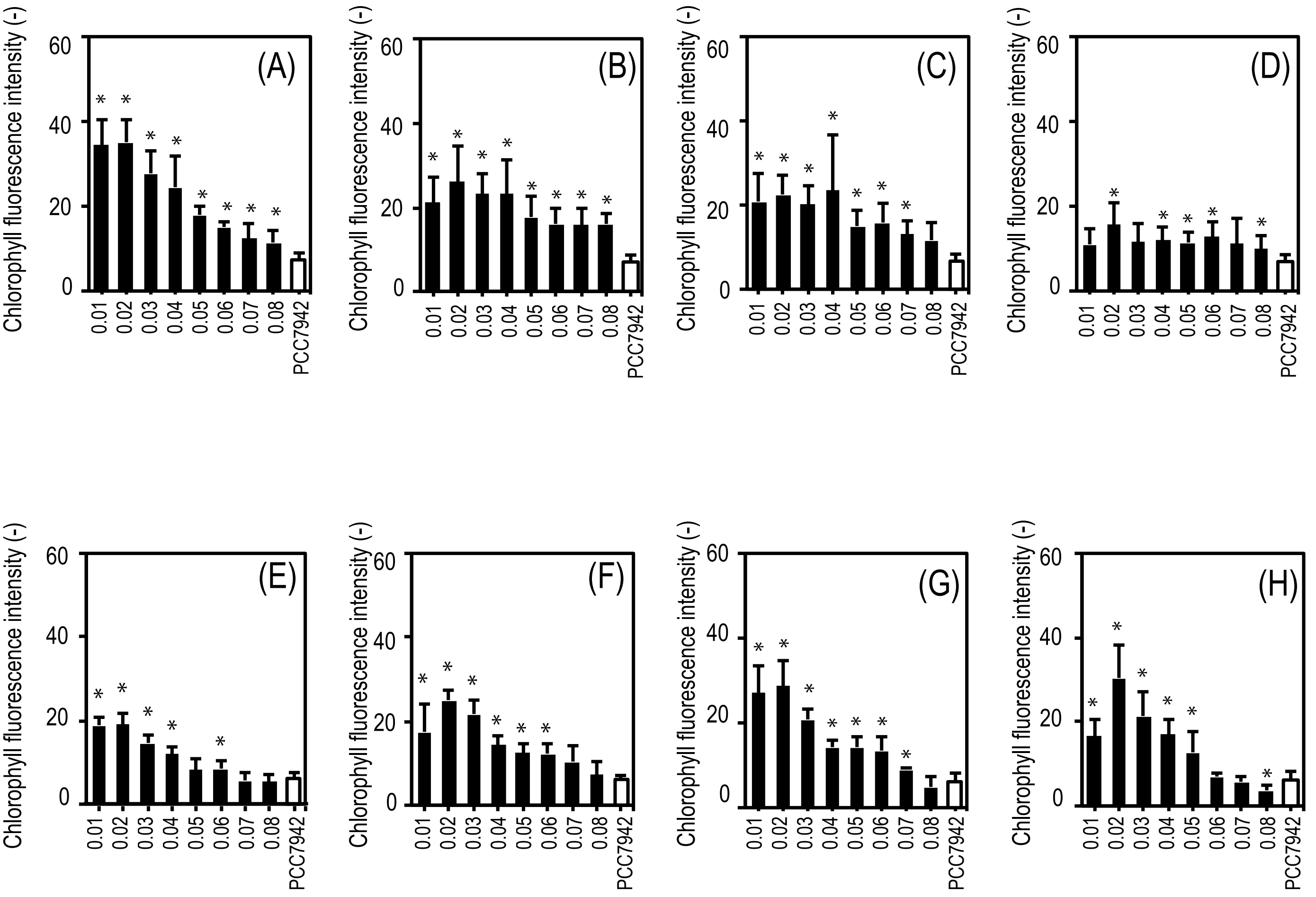

Fig. S2. Chlorophyll fluorescence intensity of *S. elongatus* PCC 7942 co-cultured with CGPBs, which prepared during different pre-culturing time periods (1 or 2 days) at different CGPB starting concentrations (optical density at 600 nm on the sixth day [x-axis]). Statistically significant differences compared with monocultured *S. elongatus* PCC 7942 are marked with an asterisk ($p < 0.05$, Student's t-test). Error bars indicate standard deviations ($n = 3$). A, co-culture with *Rhodococcus* sp. AF2108, which prepared by seed culture for 1 day; B, co-culture with *Rhodococcus* sp. AF2108, which prepared by seed culture for 2 days; C, *Ancylobacter* sp. GA1226, which prepared by seed culture for 1 day; D, *Ancylobacter* sp. GA1226, which prepared by seed culture for for 2 days; E, *Xanthobacter* sp. AF2111, which prepared by seed culture for 1 day; F, *Xanthobacter* sp. AF2111, which prepared by seed culture for 2 days; G, *Shewanella* sp. OR151, which prepared by seed culture for 1 day; H, *Shewanella* sp. OR151, which prepared by seed culture for 2 days.

(A)

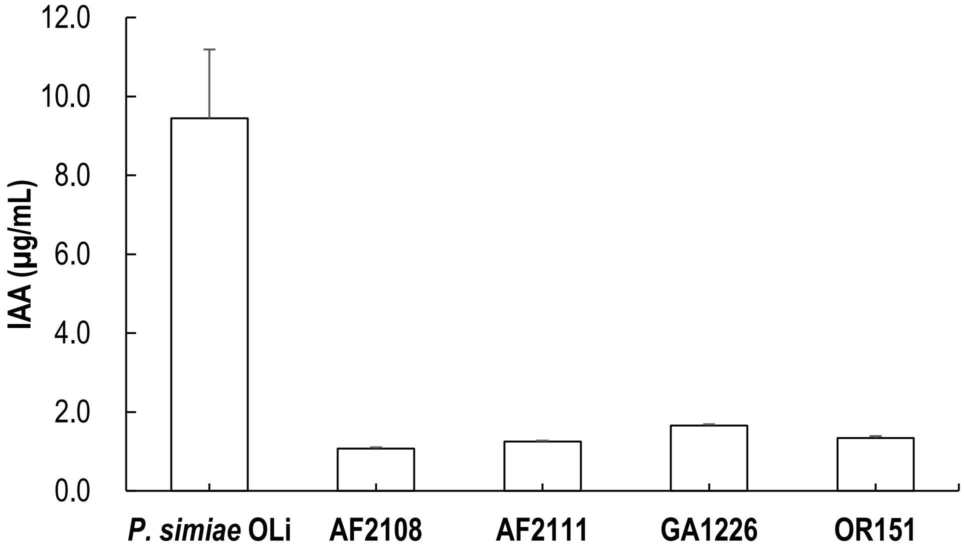

(B)

P. fluorescens (Positive control) | *Rhodococcus* sp. AF2108 | *Xanthobacter* sp. AF2108 | *Ancylobacter* sp. GA1226 | *Shewanella* sp. OR151

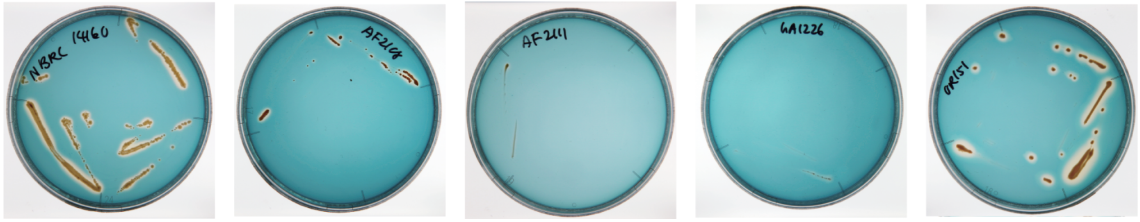

Fig. S3. Indole-3-acetic acid productivity (IAA) of the isolated strains. The error bars indicate standard deviation (n = 3). *Pseudomonas simiae* OLi was used as positive strain which can produce IAA.

(B) The results of siderophore assay using CAS blue agar. *P. fluorescens* was used as positive control.
